# Design of Digital Agricultural Extension Tools: Perspectives from Extension Agents in Nigeria

**DOI:** 10.1111/1477-9552.12371

**Published:** 2020-03-20

**Authors:** Oyakhilomen Oyinbo, Jordan Chamberlin, Miet Maertens

**Keywords:** Agricultural extension, choice experiment, digital extension technology, extension agents’ preferences, site‐specific extension, soil fertility management

## Abstract

Given the marked heterogeneous conditions in smallholder agriculture in Sub‐Saharan Africa, there is a growing policy interest in site‐specific extension advice and the use of digital extension tools to provide site‐specific information. Empirical ex‐ante studies on the design of digital extension tools and their use are rare. Using data from a choice experiment in Nigeria, we elicit and analyze the preferences of extension agents for major design features of ICT‐enabled decision support tools (DSTs) aimed at site‐specific nutrient management extension advice. We estimate different models, including mixed logit, latent class and attribute non‐attendance models. We find that extension agents are generally willing to use such DSTs and prefer a DST with a more user‐friendly interface that requires less time to generate results. We also find that preferences are heterogeneous: some extension agents care more about the effectiveness‐related features of DSTs, such as information accuracy and level of detail, while others prioritise practical features, such as tool platform, language and interface ease‐of‐use. Recognising and accommodating such preference differences may facilitate the adoption of DSTs by extension agents and thus enhance the scope for such tools to impact the agricultural production decisions of farmers.

## 1 Introduction

Traditional extension systems in Sub‐Saharan Africa (SSA) provide very general agronomic advice, such as fertiliser use recommendations, across wide and highly heterogeneous environments (Shehu *et al* *.*, 2018; Theriault *et al* *.*, 2018). Such information fails to take into account the spatial and temporal variability in biophysical and socio‐economic conditions within a given national or regional context (MacCarthy *et al* *.*, 2018; Jayne *et al.*, 2019). The use of digital decision support tools (DSTs), enabled by modern information and communication technology such as smartphones and tablets, is increasingly promoted for more effective delivery of agronomic information tailored to the site‐specific conditions of individual farmers (Bernet *et al* *.*, 2001; Kragt and Llewellyn, 2014; MacCarthy *et al* *.*, 2018). A growing number of agronomy DSTs are being developed or have recently been developed in SSA, including tools specific for maize (‘Maize‐Variety‐Selector’, ‘Maize‐Seed‐Area’), for rice (‘RiceAdvice’, ‘WeedManager’), for cassava (‘Akilimo’), for cocoa (‘CanOvaLator’) and for crops in general (‘Farmbook’, ‘Fertilizer Optimizer’, ‘FAMEWS’).

Despite the potential of DSTs to improve information delivery, their use at any sensible scale is low (Rose *et al* *.*, 2016). Constraints are posed not only by farmers who might be reluctant to take up extension advice delivered through such tools, but also by extension agents who can be reluctant to use such tools to provide extension advice to farmers (Hochman and Carberry, 2011; Ravier *et al* *.*, 2016; Rose *et al* *.*, 2016). While farmers are the ultimate recipients of DST‐supported extension advice, extension agents are most often the actual users of DSTs. Some advocate that encouraging uptake of DSTs requires design of DSTs to be driven by user‐defined preferences via a co‐design approach (Botha *et al* *.*, 2017; Ditzler *et al* *.*, 2018; Rose *et al* *.*, 2018).

In this paper, we analyse the preferences of extension agents for the design of DSTs and their willingness to use such tools. We implement a discrete choice experiment (CE) among 320 extension agents in northern Nigeria, to evaluate preferences regarding the design of a DST for site‐specific fertiliser recommendations for maize. The DST being evaluated is an implementation of the Nutrient Expert tool (Pampolino *et al* *.*, 2012), calibrated for maize in northern Nigeria. Our study provides an *ex‐ante* understanding of the potential uptake of agronomic DSTs and the specific design features that are more (or less) appealing to extension agents. Our work also generates insights on the heterogeneous preferences for the design of DSTs, and the underlying sources of heterogeneity.

Our contribution to the literature is twofold. First, to the best of our knowledge, there is only one quantitative *ex‐ante* study of extension agents’ preferences for DSTs (Kragt and Llewellyn, 2014). Our main contribution is the extension and application of this type of research. While Kragt and Llewellyn (2014) provide evidence on a DST for weed management in Australia, we build on this with evidence on a DST for nutrient management in maize farming systems in Nigeria, where the developing country context is new and potentially important. Extension agents in developing countries are more likely to be constrained in the uptake of DSTs, reflecting a lower level of education and ICT skills among extension agents; a lower level of education among recipient farmers, resulting in difficulties or more time needed to explain more detailed and more complicated extension advice to farmers. Farming conditions are highly heterogeneous in our research area, which makes nutrient management more challenging, especially for maize – a major staple crop in Nigeria and in most SSA countries. The specific application of DSTs for nutrient management advice for maize in Nigeria is different from the application of DSTs for weed management in a developed country context. Extension agents’ preferences for DSTs might also vary across locations and contexts. In addition, in comparison with Kragt and Llewellyn (2014), we use more recent data and a larger sample of respondents. Other studies on DSTs such as Rose *et al* *. *(2016, 2018) analyse the uptake of DSTs among farmers and extension agents in an *ex‐post* qualitative way, and Ditzler *et al* *. *(2018) put forward a theoretical framework to assess extension tools. Our paper complements this literature through an *ex‐ante* quantitative assessment of the preferences of extension agents for the design of DSTs.

Second, this study contributes to the CE literature by adding to the few empirical studies that implement CEs among extension agents instead of the more common use of CEs for farmers and food consumers in agricultural economics. CE studies are gaining importance in agricultural economics; they are increasingly used to assess farmers’ preferences for agricultural technologies prior to the spread of new technologies, and inform agricultural research (Breustedt *et al* *.*, 2008; Asrat *et al* *.*, 2010; Jaeck and Lifran, 2014; Lambrecht *et al* *.*, 2015; Coffie *et al* *.*, 2016; Van den Broeck *et al* *.*, 2017; Dalemans *et al* *.*, 2018; Geussens *et al* *.*, 2019). Yet, the use of CEs to inform agricultural extension *ex ante* is still very limited. Some studies use CEs to assess farmers’ preferences for extension advice from DSTs (Oyinbo *et al* *.*, 2019) but none specifically focus on extension agents except for Kragt and Llewellyn (2014). However, Kragt and Llewellyn did not account for attribute non‐attendance (ANA), a phenomenon where respondents do not consider all attributes in a CE when expressing a preference. Failure to account for ANA may lead to biased estimates (Caputo *et al* *.*, 2013; Caputo *et al* *.*, 2018). Our study extends the application of CE among extension agents with a careful consideration of ANA. The methodological issues we raise are of relevance to the design of future digital extension‐related CE research.

## 2 Research Background and Methods

### 2.1 Research area

Our study area includes three states in northern Nigeria – Kaduna, Katsina and Kano – where maize is an important staple crop (Figure A1, online Appendix). It is grown across the northern Guinea, southern Guinea and Sudan savanna agro‐ecological zones under a smallholder rain‐fed cropping system. Maize yields on farmers’ fields in the area are low, on average 1 to 2 tons per hectare despite potential yields of 5 tons per hectare or more (Shehu *et al* *.*, 2018; ten Berge *et al* *.*, 2019). A low and inappropriate use of fertiliser and other management practices contribute to low yield, and information constraints play a role in this (Shehu *et al* *.*, 2018). Traditionally, provision of extension services rests on the public sector extension systems, implemented at the state level (Naswem and Ejembi, 2017). In our study area, these are the Kaduna state agricultural development agency (KADA), the Katsina state agricultural and rural development authority (KTARDA) and the Kano state agricultural and rural development authority (KNARDA). The relatively low extension coverage of the public extension systems has given rise to other non‐governmental extension providers in recent years. Examples include increased private sector participation in the provision of advisory services (e.g., from input suppliers, agro‐dealers, etc.) as well as non‐governmental organisations such as Sasakawa‐Global 2000 (Davis and Spielman, 2017; Gizaki and Madukwe, 2019). The extension system largely provides generalised agronomic recommendations across heterogeneous locations in our study area, and in Nigeria more generally. These general recommendations are likely to be ineffective, since they fail to account for site‐specific information constraints (Naswem and Ejembi, 2017). A typical example is the provision of a general recommended fertiliser application rate of 120 kg N, 60 kg P_2_O_5_ and 60 kg K_2_O per ha for maize in much of northern Nigeria (Shehu *et al* *.*, 2018), which may well be sub‐ or supra‐optimal for the site‐specific conditions of individual farmers. The development of DSTs such as the Nutrient Expert and similar tools could enhance the capacity of the extension system, and allow the provision of site‐specific agronomic recommendations.

### 2.2 Data collection and sampling

Data were collected through a discrete choice experiment (CE) and an accompanying survey among individual extension agents in November 2016, as part of the development programme for the Nutrient Expert tool. A structured questionnaire was developed for the survey, with modules on extension agent demographics, work environment, experience with ICT, fertiliser recommendations and income sources (questionnaire is available in the online Appendix). The CE and survey were implemented using computer assisted personal interviewing, face‐to‐face with individual extension agents. Prior to this implementation, the aim of the interview and the set‐up of the CE were explained to extension agents in introductory group sessions, including all sampled extension agents in a specific survey location. We used a one‐stage random sampling design to select 320 extension agents. The study area comprises three extension institutions, including 10 governmental zonal extension offices (4 from KADA, 3 from KTARDA and 3 from KNARDA) and one major private extension provider. We randomly selected 30% of the extension agents from each of these institutions, based on a full list of frontline extension agents – i.e., extension agents who directly advise farmers in the field – provided by each of the extension offices.

### 2.3 Choice experiment design and implementation

We use a discrete CE as a stated preference elicitation method to *ex‐ante* assess extension agents’ preferences and willingness to use DSTs, before introducing the Nutrient Expert tool in the extension systems. Respondents were presented with a sequence of choice sets, each having two discrete hypothetical alternatives of a nutrient management DST, and asked to choose their most preferred alternative. The hypothetical alternatives are described by different attributes of the DST with levels that vary over the alternatives.

Based on consultations with a number of scientists involved in the development of the Nutrient Expert tool for Nigeria, a detailed review of DST design literature and a series of meetings with extension agents, we identified six relevant attributes (Table 1). These include practical attributes – level of user‐friendliness, delivery platform, delivery language and time cost – and attributes related to the content and effectiveness of the extension advice – level of detailed output and predictive power. The first attribute, ‘level of user‐friendliness’, relates to the interface ease‐of‐use of a DST, i.e., the ease of navigating through tool modules to generate extension results. The second attribute, level of detailed output, relates to the number of different recommendations that result from the DST and that need to be explained by the extension agent to the farmers as different options. Both attributes are described by three levels: low; moderate; high levels of user‐friendliness and detailed output. The third attribute ‘predictive power’ relates to the accuracy of a DST in formulating fertiliser recommendations for a farmer to achieve a certain yield. It is expressed as the percentage of farmers that can be expected to achieve the resulting yields after applying the DST‐enabled fertiliser recommendations. We include five levels ranging from less than 31% to more than 90%. The fourth attribute, ‘delivery platform’, relates to the format or platform in which extension recommendations are delivered. This is also defined by three levels: non‐mobile platforms (desktops/laptops); quick guides (paper‐based); mobile platforms (smartphones/tablets). The fifth attribute, ‘delivery language’, relates to the operating language of the tool and the recommendations. The levels are: English only, native only, and both English and native. The sixth attribute, ‘time cost’, describes the amount of time needed for an extension agent to generate a fertiliser recommendation with the DST. This attribute is defined by four levels, ranging from 15 to 60 minutes per farmer. These levels were chosen based on a possible range of time that some agents expressed as acceptable during a meeting with the extension providers.

**Table 1:** Attributes and attribute levels used in the choice experiment

Attributes	Attribute levels
User‐friendliness	Low, moderate, high
Detailed output	Low, moderate, high
Predictive power^1^	<31%, 31–50%, 51–70%, 71–90%, >90%
Delivery platform	Non‐mobile (desktops/laptops), Quick guides (paper‐based version), Mobile (smartphones/tablets)
Delivery language	English only, Native only, English + native
Time cost	15, 30, 45, 60 minutes per recommendation

Notes

^1^ We use midpoints of the attribute level ranges in the estimation. A more detailed description of the attributes and attribute levels is given in the script used to introduce and explain the CE to respondents (see the online Appendix).

We use a D‐efficient design, which minimises the number of choice sets, compared to a full factorial design, and improves the efficiency of parameter estimates (Hensher *et al* *.*, 2015). Where a pilot is not feasible, prior information, such as the expected signs, can be obtained from the empirical literature, from theory and/or from expert judgement (Rose and Bliemer, 2009). If the expected size is completely unknown, taking small priors (close to zero) with the expected signs can still allow a more efficient design over the use of null priors (Bliemer and Collins, 2016; van Cranenburgh and Collins, 2019). Based on this reasoning and on empirical applications (e.g., Dalemans *et al* *.*, 2018; Meyerhoff *et al* *.*, 2019; Van den Broeck *et al* *.*, 2017) we use small positive and negative priors (0.001 and –0.001) depending on whether we expect a positive or negative sign. These expectations were informed by discussions with some extension agents and by a review of the literature. We use Ngene software to generate the design, resulting in 12 paired choice sets randomly blocked into two blocks of six choice sets (D‐error = 0.058, A‐error = 0.255). The number of choice sets was informed by practical considerations on reducing the cognitive burden of evaluating several choice sets and allowing a minimal number of blocks to facilitate the CE implementation. From the choice sets, we constructed 12 laminated choice cards (an example is given in Figure A2 in the online Appendix) each consisting of two unlabeled hypothetical options for a nutrient management DST (options A and B) and an opt‐out (option C). An opt‐out option is included to avoid forcing the extension agents to accept the use of a DST, which corresponds to the reality of continuing to use current traditional extension methods (Hensher *et al* *.*, 2015). As described in Scarpa and Rose (2008) and implemented in Caputo *et al* *. *(2018), we report *ex‐post* efficiency measures of our design using the true parameter estimates – D‐error = 0.063, A‐error = 0.280. Taking the *ex‐ante* and *ex‐post* measures together, our design performs well with an efficiency of 92% and 91% for D‐ and A‐errors, respectively.

In the CE implementation, we started with an in group introductory session to explain the purpose of the CE, the attributes and attribute levels and the hypothetical set‐up. A cheap talk script was used to stress the need to give truthful responses and to minimise hypothetical bias (Cummings and Taylor, 1999). The same script was used for all group sessions to allow a uniform explanation across extension agents and avoid informational bias. The script is included in the online Appendix. Questions were allowed after the introduction, but we made sure our answers were only for clarification about the CE and did not prime responses. Subsequently, each agent was separately presented six choice cards in a random order by an enumerator, and was asked to choose the most preferred option. At the end of the CE, respondents were questioned about which attributes they ignored, which corresponds to serial‐based ANA, and about individual‐specific and work‐related characteristics.

## 3 Econometric Analysis

Choice experiments are rooted in random utility theory; the rationale is that utility is derived from the underlying attributes of a good or service rather than from the good or service *per se* (Lancaster, 1966) and that respondents choose those alternatives that offer the largest expected utility (McFadden, 1974). Hence, the utility $U_{\mathrm{ijs}}$ of extension agent *i* choosing alternative j in choice set s is given by an indirect utility consisting of a deterministic and a random component:

(1) $$U_{\mathrm{ijs}}=ASC+\sum_{k=1}^{6}\beta_{\mathrm{ik}}x_{\mathrm{ijks}}+\varepsilon_{\mathrm{ijs}}\;i=1,\ldots .,N;j=1,\ldots .,J;s=1,.\ldots ,S$$

The vector of attributes $x_{\mathrm{ijsk}}$ describes alternative j with associated individual‐specific parameters $\beta_{\mathrm{ik}}$ , where attribute k is an element of the vector ( k=1,…,6) . The idiosyncratic error term $\varepsilon_{\mathrm{ijs}}$ is assumed to be independently and identically distributed (iid). ASC is an alternative‐specific constant to capture preferences for the opt‐out option.

First, we estimate a mixed logit (MXL) model to account for preference heterogeneity across extension agents (Train, 2009). All parameters are specified to be random with a normal distribution. The ASC is coded as 1 for the opt‐out option, and 0 for all hypothetical DST options, which implies that a negative parameter for the ASC corresponds to a willingness to adopt DSTs. For ease of interpretation, all categorical variables are dummy‐coded.

Second, we estimate two models to account for ANA, which can be an important source of bias in the parameter estimates (Alemu *et al* *.*, 2013; Kragt, 2013; Coffie *et al* *.*, 2016). With serial stated ANA data, derived from the respondents at the end of the CE, we account for ANA in the MXL models by estimating a conventional ANA model and a validation ANA model as described in Caputo *et al* *. *(2018). In the conventional ANA method, the parameters of attributes that are reported as ignored by respondents are constrained to zero. The utility function can then be expressed as:

(2) $$U_{\mathrm{ijs}}=ASC+\sum_{k=1}^{6-\tau }\beta_{\mathrm{ik}}x_{\mathrm{ijks}}+\varepsilon_{\mathrm{ijs}}$$

where τ are attributes self‐reported as ignored. In the validation method, two parameters are estimated for each attribute depending on whether the attribute is reported as ignored or not by respondents (Alemu *et al* *.*, 2013; Scarpa *et al* *.*, 2013; Caputo *et al* *.*, 2018; Oyinbo *et al* *.*, 2019). This helps to validate the stated ANA responses and the conventional ANA model. The utility function is expressed as:

(3) $$U_{\mathrm{ijs}}=ASC+\sum_{k=1}^{6-\tau }\beta_{\mathrm{ik}}^{1}x_{\mathrm{ijks}}+\sum_{k=1}^{\tau }\beta_{\mathrm{ik}}^{0}x_{\mathrm{ijks}}+\varepsilon_{\mathrm{ijs}}$$

where, the utility coefficients conditional on attendance are indicated with the superscript 1 ( $\beta^{1}$ ) and those conditional on non‐attendance with superscript 0 ( $\beta^{0}$ ).

Third, we estimate a latent class model (LCM) to further unravel preference heterogeneity and to better explain the potential sources of heterogeneity. A LCM assumes that a heterogeneous population of extension agents consists of a discrete number of preference (or latent) classes (Hensher *et al* *.*, 2015). Preferences are assumed to be homogeneous within each latent class c but heterogeneous across classes. The probability of extension agent i choosing alternative j in choice set s is conditional on the agent’s membership of latent class c :

(4) $$P_{\mathrm{ijs}}|c=\prod_{s=1}^{S}\frac{\exp\left({\beta^{\prime }}_{c}x_{\mathrm{ijs}}\right)}{\sum_{t=1}^{J}\exp\left({\beta^{\prime }}_{c}x_{\mathrm{its}}\right)}$$

where $\beta_{c}^{{}^{\prime }}$ is the vector of class‐specific parameter estimates. The class membership probabilities are modeled using a multinomial logit with class‐specific constant terms and no respondent‐specific characteristics:

(5) $$P_{\mathrm{ic}}=\frac{\exp\left(z_{i}\right)}{\sum_{c=1}^{C}\exp\left(z_{i}\right)}$$

This implies that class membership probabilities are estimated solely taking account of the sequence of choices made by the extension agents. Respondents are then allocated to the preference classes for which they have the largest probabilities.^2^This is implemented using STATA estimation and post‐estimation commands, *lclogit* and *lclogitpr* with the *cp* option (Pacifico and Yoo, 2013). We characterise the preference classes through a comparison of means of a large set of individual‐ and work‐related characteristics of the extension agents. We follow recent empirical CE applications (e.g., Dalemans *et al* *.*, 2018; Guessens *et al*., 2019; Van den Broeck *et al* *.*, 2017) and opt for a LCM without inclusion of respondent‐specific covariates in the membership function. This allows better inferences about heterogeneous preference classes, conditioned only by the observed choice patterns, and avoids a potential bias in selecting relevant covariates in the membership function to explain observed preferences. Given that limited information is available in the literature on the preferences of extension agents and the underlying characteristics explaining these, this method suits our CE.

The joint probability of observing a sequence of choices ( $y_{\mathrm{ijs}})$ over all classes is the product of (4) and (5), and the panel formulation of the model is:

(6) $$P_{y_{\mathrm{ijs}}}=\sum_{c=1}^{C}\left[\frac{\exp\left(z_{i}\right)}{\sum_{c=1}^{C}\exp\left(z_{i}\right)}\right]\left[\prod_{s=1}^{S}\frac{\exp\left({\beta^{\prime }}_{c}x_{\mathrm{ijs}}\right)}{\sum_{t=1}^{J}\exp\left({\beta^{\prime }}_{c}x_{\mathrm{its}}\right)}\right].$$

We estimate LCMs with two to five latent classes in order to sufficiently represent preference heterogeneity in our data. Selection of the optimal number of classes is based on the Akaike Information Criteria (AIC) and the Bayesian Information Criteria (BIC) (Boxall and Adamowicz, 2002).

Fourth, to meaningfully compare the relative importance of the different attributes we need to take into account differences in scale (Greene and Hensher, 2003). To this end, we estimate marginal rates of substitution (MRS) between time cost and other attributes using the Krinsky‐Robb method with 2000 draws (Krinsky and Robb, 1986). The MRS is interpreted as the willingness to accept a higher time cost and by extension, more effort in the use of a DST for an increase in the utility of another attribute. The MRS estimation is based on the results of the LCM, as this model allows a better interpretation in terms of differences in the magnitude of trade‐offs between time costs and other attributes across the classes of extension agents.

## 4 Results

Table 2 describes the individual‐ and work‐related characteristics of the extension agents, which are used to explain observed preference heterogeneity (as described below). The large majority (95%) of the extension agents are male. Their average age is 40 years, and they had an average of almost 19 years of schooling. About three quarters of the extension agents report to be proficient in the use of smartphones and/or tablets but only 44% own a smartphone and only 2% own a tablet. Also, the majority (87%) is affiliated to the public extension system. The average extension experience in the sample is 12.7 years. About 30% of the extension agents have ICT‐based extension experience and 72% report being aware of site‐specific nutrient management advice. Seventy two percent and 21% report having received training on, respectively, soil fertility issues and ICT aspects in the last 12 months prior to the survey. Only 48% of the extension agents have access to a vehicle, motorcycle or bicycle to carry out their extension work. More than 90% of the extension agents agree that they receive adequate supervision and timely remuneration, and more than 80% receive regular promotion.

**Table 2:** Summary statistics of extension agents’ characteristics

Variables	Description of variables	Mean	Std. Dev.
Individual‐specific characteristics			
Male (yes = 1)	Gender of extension agent	0.95	0.35
Age (years)	Age of extension agent	39.58	10.48
Married (yes = 1)	Marital status of extension agent	0.82	0.39
Education (years)	Years of schooling attained	18.88	1.82
Engage in agriculture (yes = 1)	Self‐reported engagement in agriculture	0.88	0.32
Proficient in the use of a smartphone/tablet (yes = 1)	Self‐assessed proficiency in the use of a smartphone/tablet	0.74	0.44
Own a smartphone (yes = 1)	Ownership of a smartphone	0.44	0.50
Own a tablet (yes = 1)	Ownership of a tablet	0.02	0.15
Work‐related characteristics			
Affiliated to public extension (yes = 1)	Affiliation of extension agent	0.87	0.34
Extension experience (years)	Years of working in the extension system attained by extension agent	12.74	10.27
ICT‐based extension experience (yes = 1)	Self‐reported experience on the use of digital technologies, such as smartphones and tablets for extension purposes	0.29	0.45
Soil fertility‐related training (yes = 1)	Self‐reported access to training on soil fertility the past one year	0.72	0.45
ICT‐related training (yes = 1)	Self‐reported access to training on ICT in the past one year	0.21	0.41
Access to transport facilities (yes = 1)	Self‐reported access to transport facilities, such as bicycle, motorcycle or vehicle for extension purposes	0.48	0.49
Receive adequate supervision (yes = 1)	Self‐assessed adequacy of supervision	0.95	0.22
Receive regular promotion (yes = 1)	Self‐assessed regularity of promotion	0.83	0.38
Receive timely remuneration (yes = 1)	Self‐assessed timeliness of remuneration	0.96	0.20
Perceive job to be secure (yes = 1)	Self‐assessed job security	0.93	0.25
% of working time devoted to soil fertility‐related issues (%)	Self‐estimated share of time devoted to soil‐fertility related issues	63.22	
Aware of site‐specific nutrient management (yes = 1)	Self‐reported awareness of site‐specific nutrient management	0.72	0.45
Farmers often request soil fertility‐related advice (yes = 1)	Self‐reported farmers’ request for soil fertility‐related advice	0.98	0.16
Observations		320	

Table 3 reports the results of the mixed logit (MXL) models, including the standard MXL without controlling for ANA, the conventional ANA and validation ANA models. Thirty three percent of the extension agents report ignoring at least one attribute, which supports the estimation of ANA models. The estimated conventional ANA model is qualitatively similar to the standard MXL model in terms of expected signs of the coefficients, while the model fit using the AIC and BIC information criteria is similar across the models, implying that our results are robust to potential ANA bias.^3^One cannot compare the magnitude of coefficients between models because of scale differences (Greene and Hensher, 2003), and so we make no claim about similarity in terms of magnitude of coefficients. This is corroborated by the results of the validation ANA model, which show that coefficients for ignored attributes are not significantly different from zero – except for predictive power. This implies that the choice behaviour of the extension agents is consistent with their stated ANA information, and that hence the conventional ANA and the MXL results are not biased due to ANA (Scarpa *et al* *.*, 2013; Caputo *et al* *.*, 2018). Overall, the ANA models do not clearly outperform the MXL model. A plausible explanation for this relates to the fact that individual respondents ignore only a few attributes and the ignored attributes vary in the sample (Table A1 in the online Appendix). This may also reflect limitations related to ‘measurement errors*’* in serial ANA models, as mentioned by Caputo *et al* *. *(2018). Therefore we base our discussion on the MXL results.

**Table 3:** Results of mixed logit models, with and without control for attribute non‐attendance (ANA)

	MXL		Conventional ANA		Validation ANA			
					Considered attributes		Ignored attributes	
	Mean	Std. Dev.	Mean	Std. Dev.	Mean	Std. Dev.	Mean	Std. Dev.
ASC	−3.41***, ***	1.37**	−3.78***, ***	1.55***, ***	−3.59***, ***	1.60***, ***		
	(0.61)	(0.54)	(0.64)	(0.51)	(0.69)	(0.59)		
Time cost (minutes/output)	−0.01**	−0.01*	−0.01***, ***	0.01***, ***	−0.01**	0.01**	−0.01	0.00
	(0.00)	(0.01)	(0.00)	(0.00)	(0.00)	(0.01)	(0.01)	(0.02)
User‐friendliness: moderate	0.48***, ***	0.55***, ***	0.56***, ***	0.58***, ***	0.49***, ***	0.60***, ***	0.53	0.09
	(0.12)	(0.17)	(0.12)	(0.18)	(0.13)	(0.18)	(0.36)	(0.59)
User‐friendliness: high	0.47***, ***	0.28	0.55***, ***	0.26	0.50***, ***	0.25	0.29	0.47
	(0.11)	(0.39)	(0.11)	(0.32)	(0.12)	(0.39)	(0.39)	(0.69)
Detailed output: moderate	0.34***, ***	−0.03	0.28***, ***	0.24	0.37***, ***	0.26	−0.13	0.06
	(0.10)	(0.47)	(0.10)	(0.33)	(0.11)	(0.34)	(0.45)	(0.63)
Detailed output: high	0.28***, ***	0.29	0.29***, ***	0.30	0.28**	0.41*	0.57	0.14
	(0.10)	(0.33)	(0.10)	(0.31)	(0.11)	(0.24)	(0.46)	(1.51)
Predictive power	0.01***, ***	−0.01***, ***	0.01***, ***	0.00	0.01***, ***	0.01*	0.01***, ***	0.02***, ***
	(0.00)	(0.00)	(0.00)	(0.00)	(0.00)	(0.00)	(0.01)	(0.01)
Platform: paper	−0.20**	0.71***, ***	−0.21*	0.80***, ***	−0.23**	0.90***, ***	0.06	0.02
	(0.10)	(0.17)	(0.11)	(0.17)	(0.11)	(0.17)	(0.37)	(0.37)
Platform: mobile	0.40***, ***	−0.36	0.38***, ***	0.31**	0.41***, ***	0.40*	0.53	0.58
	(0. 09)	(0.22)	(0.09)	(0.26)	(0.09)	(0.23)	(0.36)	(0.57)
Language: native	0.19*	−0.20	0.20*	0.22	0.23*	0.15	−0.25	0.40
	(0.10)	(0.29)	(0.10)	(0.27)	(0.11)	(0.36)	(0.35)	(0.68)
Language: English + native	0.36***, ***	0.79***, ***	0.32**	0.84***, ***	0.43***, ***	0.92***, ***	−0.09	0.00
	(0.14)	(0.17)	(0.13)	(0.16)	(0.15)	(0.17)	(0.38)	(0.70)
N	5,760		5,760		5,760			
Log likelihood	−1,348.12		−1,354.12		−1,333.95			
AIC	2,740.25		2,752.20		2,751.90			
BIC	2,886.75		2,874.60		2,985.40			

Notes

Standard errors reported in parentheses.

*** ^,^ ** and * denote any variable significant at 1%, 5% and 10% levels respectively.

The ASC coefficient estimate is significantly negative, which indicates that the extension agents generally prefer the use of DSTs for site‐specific extension advice on nutrient management. This supports the ongoing efforts to develop such DSTs for maize in the research area. In general, the extension agents prefer DSTs with a higher level of user‐friendliness, more detailed output, and a higher predictive power. In addition, they prefer a mobile platform in the native language, or a combination of English and the native language. The extension agents disliked DSTs that have a higher time demand per output and paper‐based DST platforms. The standard deviations are statistically significant for most of the attributes. This implies that there is preference heterogeneity across extension agents, although the large majority prefer DSTs with lower time demand (84%), with moderate user‐friendliness (81%), with higher predictive power (84%), with both English and the native language (68%), and dislike paper‐based platforms (61%). We observe that the preferences between agents with and without access to smartphones only vary slightly (Table A2 in the online Appendix). Also, we find no major differences in preferences between agents across the three study states (Table A3 in the online Appendix). Nevertheless, we look beyond the general findings and consider distinct sub‐groups of agents defined by their choice behaviour via a LCM, to provide more insight into the preference heterogeneity and identify any practical implications for DST design and targeting.

Table 4 presents the results of a latent class model. We selected a model with two latent classes based on a comparison of the information criteria across models with from two to five classes (Table A4 in the online Appendix). Preference class one (PC1) includes 52% of the sampled extension agents and preference class 2 (PC2) 48%. In both classes, extension agents are in general willing to accept the use of DSTs, and have strong preferences for DSTs that limit the time demand per recommendation output and that have a moderately to highly user‐friendly interface. Yet, we observe substantial heterogeneity in preferences between the two classes for the other attributes. Extension agents of PC1 prefer DSTs with highly detailed output and a strong predictive power while those in PC2 are indifferent to these attributes. Extension agents of PC2 prefer DSTs on mobile devices, that use the native language or a combination of the native language and English, and dislike paper‐based tools while those in PC1 are indifferent to these attributes.

**Table 4:** Results of latent class models

	Preference class 1 = 52%		Preference class 2 = 48%	
	Coefficient	Std. error	Coefficient	Std. error
ASC	−2.15***, ***	0.40	−3.66***, ***	0.93
Time cost (minutes/output)	−0.01*	0.00	−0.01*	0.01
User‐friendliness: moderate	0.39**	0.18	0.67***, ***	0.25
User‐friendliness: high	0.19	0.18	1.02***, ***	0.31
Detailed output: moderate	0.26	0.18	0.35	0.25
Detailed output: high	0.45***, ***	0.16	−0.12	0.28
Predictive power	0.01***, ***	0.00	0.00	0.00
Platform: paper	0.21	0.19	−0.66*	0.35
Platform: mobile	0.15	0.15	0.59***, ***	0.15
Language: native	0.15	0.16	0.46*	0.25
Language: English + native	−0.19	0.27	1.14**	0.56
N	5,760			
Log likelihood	−1,344.04			
AIC	2,734.07			
CAIC	2,910.23			
BIC	2,887.22			

Notes

*** ^,^ ** and * denote any variable significant at 1%, 5% and 10% levels, respectively.

To explore the sources of preference heterogeneity, we compare individual‐ and work‐related characteristics of extension agents between the two PCs (Table 5). The results show that PC2 extension agents have a significantly higher education, a lower likelihood of engagement in agriculture, and a higher likelihood to be proficient in the use of smartphones and/or tablets, to have experience with ICT‐based extension, to receive regular promotion and to be paid in a timely manner. This might explain their strong preferences for DSTs with mobile platforms. Yet, they appear to care less about the level of detail and accuracy of extension advice, which suggests that the appeal for these attributes is not necessarily correlated with education and ICT proficiency of extension agents, or with receipt of timely remuneration and regular promotion. Overall, the differences in observed characteristics between the two PCs are significant but very small, which implies that unobservable characteristics, such as motivation and ability, likely play a role as well in determining preference heterogeneity.

**Table 5:** Profile of extension agent characteristics across latent preference classes

	PC1 = 52%		PC2 = 48%		
	Mean	Std. Dev.	Mean	Std. Dev.	Sig.
Individual‐specific characteristics					
Male	0.95		0.95		
Age	39.64	10.49	39.51	10.49	
Married	0.79	0.41	0.85	0.36	
Education	18.71	1.88	19.06	1.73	*
Engage in agriculture	0.91	0.29	0.85	0.36	*
Proficient in the use of a smartphone/tablet	0.70	0.46	0.78	0.41	*
Own a smartphone	0.40	0.49	0.48	0.50	
Own a tablet	0.01	0.11	0.03	0.18	
Work‐related characteristics					
Affiliated to public extension	0.87	0.33	0.86	0.35	
Extension experience	12.78	10.45	12.69	10.09	
ICT‐based extension experience	0.23	0.42	0.35	0.48	**
Soil fertility‐related training	0.75	0.44	0.69	0.46	
ICT‐related training	0.19	0.39	0.24	0.42	
Access to transport facilities	0.49	0.50	0.46	0.50	
Receive adequate supervision	0.95	0.23	0.95	0.22	
Receive regular promotion	0.80	0.40	0.87	0.34	*
Receive timely remuneration	0.93	0.25	0.98	0.14	**
Perceive job to be secure	0.94	0.24	0.93	0.26	
% of working time devoted to soil fertility‐related issues	63.0		63.5		
Aware of site‐specific nutrient management	0.74	0.44	0.69	0.46	
Farmers often request soil fertility‐related advice	0.98	0.13	0.97	0.18	

Notes

Two‐sided *t‐*tests of mean differences between extension agents in PC1 and 2.

** and * denote significant differences at 5% and 10% levels, respectively. Variables are as described in Table 2.

Table 6 reports the estimated MRS between time cost and other attributes. The MRS estimates show that in both classes extension agents are willing to accept a higher time cost for a more user‐friendly interface, but this trade‐off is on average larger in PC2. In addition, extension agents in PC1 are willing to accept a higher time cost for a more detailed and more accurate output while extension agents in PC2 are not. The latter are willing to accept a higher time cost for a mobile delivery platform in the native language, or a combination of English and the native language. This is plausible since some of the extension agents may have limited English language proficiency.

**Table 6:** Marginal rate of substitution (MRS) between time cost and other attributes^1^, ^2^

	Preference class 1	Preference class 2
	Mean (95% confidence interval)	Mean (95% confidence interval)
User‐friendliness: moderate	66.86 (−313.20–498.99)	74.31 (−203.49–360.52)
User‐friendliness: high	33.37^1^, ^2^ (−92.89–283.70)	113.42 (−193.98–465.70)
Detailed output: moderate	45.19^1^, ^2^ (−123.32–256.86)	38.78^1^, ^2^ (−55.37–144.69)
Detailed output: high	78.84 (−254.35–489.48)	–13.10^1^, ^2^ (−144.38–93.13)
Predictive power	1.45 (−7.20–10.55)	0.02^1^, ^2^ (−1.37–1.49)
Platform: paper	36.75^1^, ^2^ (−164.65–316.66)	–73.51 (−295.43–91.49)
Platform: mobile	26.28^1^, ^2^ (−82.00–180.73)	65.50 (−147.91–288.33)
Language: native	26.48^1^, ^2^ (−110.71–203.32)	51.20 (−52.04–204.79)
Language: English + native	–32.54^1^, ^2^ (−349.82–203.83)	126.41 (−106.89–467.53)

Notes

^1^ Estimated based on coefficients in the latent class model in Table 4,

^2^ Not discussed as coefficients are insignificant.

## 5 Discussion and Conclusion

We find that extension agents in the maize belt of Nigeria are in general willing to accept the use of DSTs for site‐specific extension services on nutrient management for maize. While extension agents in the sample prefer DSTs with a more user‐friendly interface that require less time to generate an output, we observe substantial preference heterogeneity for the other design features of DSTs, and identify two groups of extension agents with different preference patterns. Extension agents in PC1 (52%) care more about attributes related to the effectiveness of the extension advice resulting from a DST, such as a more detailed and more accurate output. These extension agents are more likely to be motivated to use DSTs through a careful explanation of the underlying science and evidence‐based aspects of DSTs. Extension agents in PC2 (48%) care more about the practical attributes of DSTs such as the platform, the language and the user‐friendliness of the interface. These extension agents are more likely to be easily convinced about the use of DSTs if the practical and operational aspects of DSTs are taken care of. Reflecting on the sources of heterogeneous preferences, the role of observed characteristics is quite small and hence, unobservable characteristics, e.g., motivation and ability, are likely to play a role in explaining the differences in preferences. However, we cannot analyse the role of motivation and ability in more depth as we do not have proxy variables for these typically unobservable characteristics in our data.

Our finding that extension agents prefer DSTs with a user‐friendly interface and a lower time requirement is partly consistent with Kragt and Llewellyn (2014), who report preferences for low time cost in a weed management DST in a developed country context but did not consider interface ease‐of‐use. In addition, our results are in line with the extant literature on the design of user‐friendly interfaces to stimulate the use of such tools (Bernet *et al* *.*, 2001; Hochman and Carberry, 2011; Rose *et al* *.*, 2016). Our finding of a strong preference by the extension agents in PC2 for DSTs on mobile devices such as smartphones and tablets contrasts with Kragt and Llewellyn (2014), who found extension agents in their sample to prefer a spreadsheet‐based platform. The result that some extension agents prefer the use of native or a combination of native and English language is consistent with Tata and McNamara (2016), who suggest that the use of local languages in the design of ‘*farmbook’*, an ICT‐based extension tool, is more beneficial to farmers. This will likely facilitate better communication with the majority of farmers who do not understand English, and reduces the likelihood of misinterpreting the inputs and outputs of DSTs. Our findings on the strong preferences of extension agents for DSTs that provide a more accurate and more detailed output are consistent with some studies that considered these attributes. For example, Kragt and Llewellyn (2014) find that a DST that generates more accurate output is strongly desired across the groups of extension agents identified in their study, whereas we find this only to be the case for the extension agents in PC1. Qualitatively, Hochman and Carberry (2011) find that the use of DSTs that allow the provision of a wide range of options to farmers is well liked by tool users in a developed country setting. The fact that the sources of observed heterogeneous preferences in our study appear to derive from unobservable characteristics is consistent with Kragt and Llewellyn (2014) who find that observed demographic characteristics were not significant in explaining preferences.

We provide some specific policy implications of our findings. Our results imply that there is high potential demand for ICT‐enabled DSTs for site‐specific extension services in our study area – a finding which aligns with the currently widespread interest and investments in site‐specific and ICT‐enabled extension tools for agricultural applications in developing countries. Our results imply that a user‐friendly interface and a reduced time effort needed to generate extension advice are important design features for a DST. To stimulate uptake and facilitate better targeting, a more effective design will likely require DSTs to be differentiated along dimensions of their practical attributes such as the platform and the language. However, differentiating DSTs according to effectiveness attributes is probably unacceptable as this would result in quality differentiation among farmers of the extension advice they receive. The effectiveness of the advice should be strongly considered in the design stages of DSTs to provide higher‐quality agronomic advice to all farmers. Extension agents who are indifferent to DSTs that can offer a more accurate and more detailed output – i.e., those in PC2 – may need to be better disposed to the quality of extension advice from DSTs beyond the practical features of DSTs. This may require improved capacity building for such agents (Davis and Spielman, 2017; Makate and Makate, 2019).

In terms of methodological issues, our work engages with the growing scholarly interest on ANA in the CE literature. While we account for ANA using a serial stated ANA approach and do not find significant improvements in model fit, future CEs among extension agents in developing countries can explore other approaches less prone to measurement errors, such as choice task stated ANA, eye tracking and inferred ANA.

Finally, our empirical findings have direct implications for the development of the nutrient management DST for maize ‘Nutrient Expert’ in Nigeria.^4^Unfortunately, the institution responsible for developing the tool in this project – the International Plant Nutrition Institute (IPNI) – ceased operations in April 2019. A new institution – the African Plant Nutrition Institute (APNI) – has been created to build on the IPNI’s plant nutrition research and education in Africa. It is not yet clear to what extent or how APNI will continue development of the Nutrient Expert tool for maize in Nigeria. Our findings have contributed to informing the choice of delivery language and selection of the tool delivery platform among propositions for a paper‐based platform, quick guides and other possible platforms. Our results show that tool user‐friendliness and the time required to generate farm‐specific advice are acutely important for extension agents. The practical implication is that DST development should consider time optimization – for example through fine tuning the color‐text‐image combinations of the interface of the tool and directly engaging extension agents in testing of interface alternatives, and identifying the specific amount of time that is acceptable for the use of the tool in a given context. In addition, the engagement of extension agents is required in testing variants of DST outputs with varying levels of detail in the output to optimise a DST in line with extension agents’ preferences. An attractive DST should not only optimise the output in terms of accurate nutrient management advice but also balance this with optimising user‐ and convenience‐related features to ensure wide uptake.

## Supporting information


**Appendix A1** **.** Script for implementation of choice experiment
**Figure A1** **.** Map of the study area
**Figure A2** **.** Example of a choice card used in the choice experiment
**Table A1** **.** Self‐reported information on ANA – serial stated ANA.
**Table A2** **.** Results of MXL models showing heterogeneity in preferences for DST features by access to smartphones.
**Table A3** **.** Results of MXL models showing heterogeneity in preferences for DST features by states where extension agents work in the research area.
**Table A4** **.** Criteria for the selection of optimal number of preference classes (N = 5,760).
**Survey questionnaire** **.** Taking Maize Agronomy to Scale in Africa (TAMASA) Extension Agents Survey 2016.
