# Supplementary material for: Design of Digital Agricultural Extension Tools: Perspectives from Extension Agents in Nigeria
Source: J Agric Econ. 2020 Mar 20;71(3):798–815. doi: 10.1111/1477-9552.12371 (PMC7508041; doi:10.1111/1477-9552.12371)
Supplement: Supplementary file 1 — Appendix A1 . Script for implementation of choice experiment Figure A1 . Map of the study area Figure A2 . Example of a choice card used in the choice experiment Table A1 . Self‐reported information on ANA – serial stated ANA. Table A2 . Results of MXL models showing heterogeneity in preferences for DST features by access to smartphones. Table A3 . Results of MXL models showing heterogeneity in preferences for DST features by states where extension agents work in the research area. Table A4 . Criteria for the selection of optimal number of preference classes (N = 5,760). Survey questionnaire . Taking Maize Agronomy to Scale in Africa (TAMASA) Extension Agents Survey 2016. [file JAGE-71-798-s001.docx]

**Design of Digital Agricultural Extension Tools: Perspectives from** **Extension Agents in Nigeria**

Oyakhilomen Oyinbo, Jordan Chamberlin and Miet Maertens

**ON-LINE APPENDIX**

**Design of Digital Agricultural Extension Tools: Perspectives from** **Extension Agents in Nigeria**


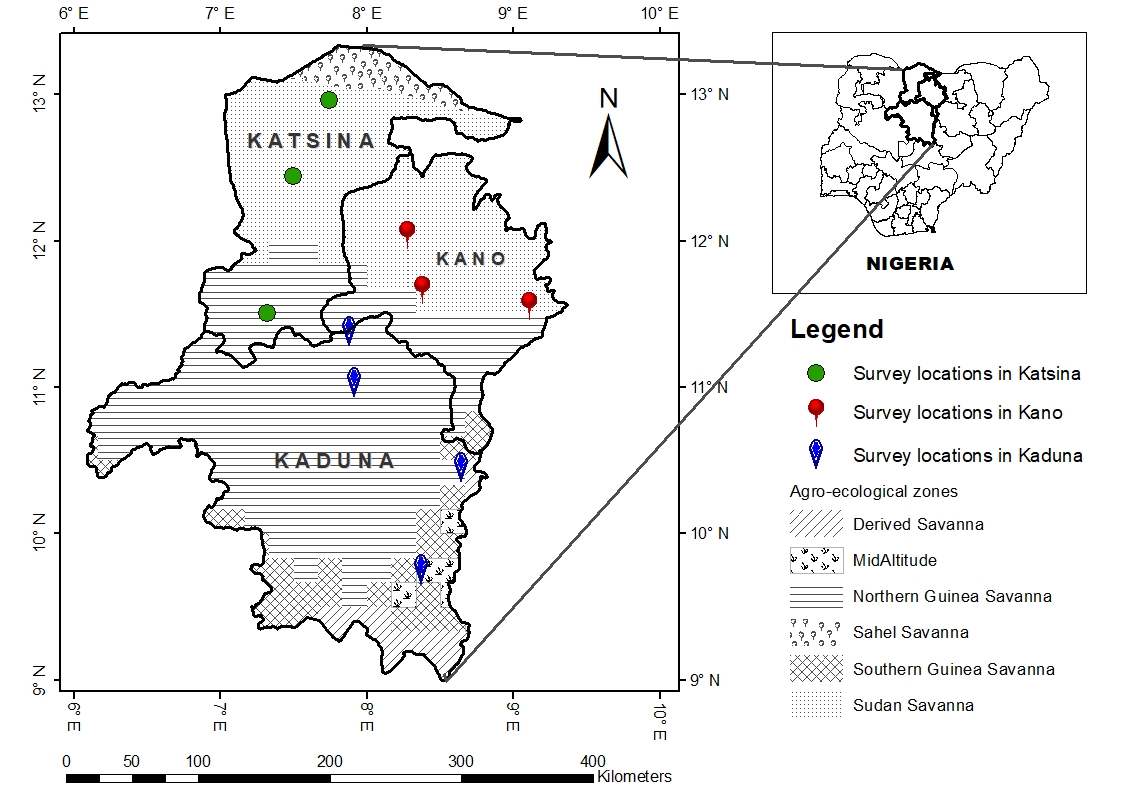


Figure A1: Map of the study area

Figure A2. Example of a choice card used in the choice experiment

**Appendix A: Script for implementation of choice experiment**

In a bid better serve farmers with SITE-SPECIFIC extension services in which recommendations are tailored to site-specific conditions of individual farmers instead of the conventional GENERAL extension recommendations, your inputs are highly needed to optimize the design of a nutrient management decision support tool for maize ‘‘Nutrient Expert”. The design is expected to result in an extension tool that can enable you to provide site-specific nutrient management (SSNM) recommendations to farmers, which will help them make better informed decisions on soil fertility and crop management. We (referring to supervisors and enumerators) will guide you through an exercise in which you will have the opportunity to choose different hypothetical options of nutrient management decision support tools for maize, and the options will be presented in the form of a card called choice card. These options are defined using six features called attributes, namely level of user-friendliness, level of detailed output, predictive power, delivery platform, delivery language and time cost. Each of the six attributes have different levels called attribute levels (At this point, the attributes and attribute levels are described in detail).

**Description of attributes**

1. **Level of user-friendliness**

This attribute relates to the user-interface of a nutrient management DST and the ease of navigating through modules of the tool (i.e. the necessary steps) to generate an extension output for a farmer. In other words, it describes the ease with which an extension agent can interact with a tool via the interface. This is defined by three levels, namely low, moderate and high levels of user-friendliness where a high level of user-friendliness is rated above a moderate level, and a moderate level is rated above a low level. To distinguish between the three levels in a choice card, an image showing only a command-line interface, a combination of command-line and graphical user interfaces and only a graphical user interface will be used to depict low, moderate and high levels of interface ease-of-use respectively. At this point, a sample of a card that shows the three levels is presented to the extension agents to enable them see in practice how the attribute levels will be depicted in the choice cards.

1. **Level of detailed output**

This attribute relates to the number of different recommendations that result from a nutrient management DST and that should be explained by an extension agent to a farmer as different options of fertilizer use recommendations. In other words, the level of information set that can be produced from a DST for a farmer. This is described by three levels, namely low, moderate and high levels of detailed output where a high level of detailed output is rated above a moderate level, and a moderate level is rated above a low level. To distinguish between the three levels in a choice card, an image with a larger portion being blurred (smaller portion with text), halfway blurred and halfway with text, and all portion with text will be used to depict low, moderate and high levels of interface detailed output from a DST respectively. At this point, a sample of a card that shows the three levels is presented to the extension agents to enable them see in practice how the attribute levels will be depicted in the choice cards.

1. **Predictive power**

This attribute relates to the accuracy of a DST in formulating fertilizer recommendations for a farmer to achieve a certain expected yield. It is expressed as the percentage of farmers that can achieve expected yields after applying the DST-enabled fertilizer recommendations received from extension agents. This is defined by five levels, < 31%, 31 – 50%, 51 – 70%, 71 – 90% and > 90% where < 31% indicates the % of farmers who realize the expected yields associated with a fertilizer use recommendation from a DST. The same interpretation applies to 31 – 50%, 51 – 70%, 71 – 90% and > 90%. To distinguish between the five levels in a choice card, different versions of an image of a group of farmers with different extent of blurry portions as a sign of differences in realizing the expected yields will be used to depict the different levels of the predictive power of a DST. At this point, a sample of a card that shows the five levels is presented to the extension agents to enable them to see in practice how the attribute levels will be depicted in the choice cards.

1. **Delivery platform**

This attribute relates to the format or platform in which extension recommendations are delivered to farmers from a nutrient management DST. This is defined by three levels, including the use of non-mobile platforms, such as desktop and laptop computers, the use of quick guides, i.e. paper-based platforms, and the use of mobile platforms, such as smartphones and tablets. To distinguish between the three levels in a choice card, an image of desktop and laptop computers, an image of a paper extension guide and an image of smartphones and tablets will be used to depict the three levels of a delivery platform. At this point, a sample of a card that shows the three levels is presented to the extension agents to enable them see in practice how the attribute levels will be depicted in the choice cards.

1. **Delivery language**

This attribute relates to the operating language of a nutrient management DST and the recommendation output of the DST. This is defined by three levels, namely the use of English language only, the use of native language only, and a combination of English and native language. To distinguish between the three levels in a choice card, an image showing some text in only English language, in only native language and in both English and native languages will be used to depict the three levels of a DST delivery language. At this point, a sample of a card that shows the three levels is presented to the extension agents to enable them see in practice how the attribute levels will be depicted in the choice cards.

1. **Time cost**

This attribute describes the amount of time needed for an extension agent to generate a fertilizer recommendation with a nutrient management DST. This is defined by four levels, namely 15, 30, 45, 60 minutes per recommendation from a DST. To distinguish between the four levels in a choice card, an image showing the different amount of time in minutes will be used to depict the four levels of time cost of using a nutrient management DST to offer extension advice to a farmer. At this point, a sample of a card that shows the four levels is presented to the extension agents to enable them see in practice how the attribute levels will be depicted in the choice cards.

After the group introductory session, we will have a face-to-face interview with each extension agent. In the interview, each agent will be offered six distinct choice cards one after the other and each choice card contains two hypothetical scenarios of nutrient management decision support tools (options A and B) and a third option (option C) that reflects your current extension approach. The aim is for you to choose one option that you prefer from the three options on each card, and this will require you to objectively reflect on the attribute levels of the two hypothetical scenarios of nutrient management decision support tools in comparison with your extension approach. You are to carefully go through the cards and evaluate the options on each card that we will present to you, and then select the option you prefer between the three options on each of the cards. Even though this exercise entails hypothetical options of nutrient management decision support tools, you are expected to kindly make very truthful choices as if these were real choices that have real cost implications. This is to ensure that the choices you make in this hypothetical exercise are not different from the actual choices if you were exposed to real nutrient management decision support tools. At this point, a sample of a choice card is shown to the extension agents with a description of the rows and columns of the card.

Table A1

Self-reported information on ANA – serial stated ANA

| # of ignored attributes | Share of extension  agents (%) | Ignored attributes | Share of extension agents (%) |
| --- | --- | --- | --- |
| 0 | 67.5 | Level of user-friendliness | 6.9 |
| 1 | 22.2 | Level of detailed output | 3.8 |
| 2 | 9.4 | Predictive power | 13.7 |
| 3 | 0.9 | Delivery platform | 5.6 |
|  |  | Delivery language | 6.6 |
|  |  | Time cost | 7.2 |

Table A2

Results of MXL models showing heterogeneity in preferences for DST features by access to smartphones

|  | Agents with smartphones | | Agents without smartphones | |
| --- | --- | --- | --- | --- |
|  | Mean | Std. Dev. | Mean | Std. Dev. |
|  |  |  |  |  |
| ASC | -4.93***  (1.44) | 2.46**  (1.08) | -2.7***  (0.73) | -0.86  (0.86) |
| Time cost (minutes/output) | -0.01***  (0.00) | 0.02**  (0.01) | -0.00  (0.00) | -0.00  (0.00) |
| User-friendliness: moderate | 0.81***  (0.27) | 0.99***  (0.34) | 0.38**  (0.16) | 0.62***  (0.21) |
| User-friendliness: high | 0.91***  (0.24) | -0.37  (0.37) | 0.28*  (0.15) | -0.50*  (0.27) |
| Detailed output: moderate | 0.39*  (0.22) | 1.01***  (0.39) | 0.39***  (0.14) | -0.11  (0.37) |
| Detailed output: high | 0.34*  (0.20) | -0.41  (0.36) | 0.26*  (0.15) | -0.37  (0.34) |
| Predictive power | 0.01**  (0.00) | 0.01***  (0.00) | 0.01***  (0.00) | 0.01**  (0.00) |
| Platform: paper | -0.60**  (0.24) | 0.97***  (0.32) | -0.07  (0.14) | 0.74***  (0.23) |
| Platform: mobile | 0.60***  (0.20) | -0.61*  (0.33) | 0.40***  (0.12) | 0.47*  (0.24) |
| Language: native | 0.07  (0.20) | -0.82**  (0.29) | 0.28**  (0.14) | 0.12  (0.34) |
| Language: English+ native | 0.25  (0.26) | 1.21***  (0.04) | 0.51***  (0.19) | -0.78***  (0.21) |
| N | 2520 |  | 3240 |  |
| Log likelihood | -571.81 |  | -763.52 |  |
| AIC | 1187.61 |  | 1571.03 |  |
| BIC | 1315.92 |  | 1704.86 |  |

***Notes***: Asterisks ***, **, and * denote any variable significant at 1%, 5%, and 10% levels respectively.

Standard errors reported between parentheses.

Table A3

Results of MXL models showing heterogeneity in preferences for DST features by states where extension agents work in the research area

|  | Agents in Kaduna | |  | Agents in Katsina | |  | Agents in Kano | |
| --- | --- | --- | --- | --- | --- | --- | --- | --- |
|  | Mean | Std. Dev. |  | Mean | Std. Dev. |  | Mean | Std. Dev. |
| ASC | -3.52*** | -0.72 |  | -2.15*** | 0.19 |  | -4.12*** | -2.04*** |
|  | (0.76) | (0.94) |  | (0.66) | (1.36) |  | (1.11) | (0.69) |
| Time cost (minutes/output) | -0.02*** | 0.02** |  | -0.00 | 0.01 |  | -0.00 | 0.00 |
|  | (0.01) | (0.01) |  | (0.00) | (0.01) |  | (0.00) | (0.01) |
| User-friendliness: moderate | 0.67** | 0.66 |  | 0.52** | 0.57 |  | 0.45** | 0.71*** |
|  | (0.26) | (0.42) |  | (0.26) | (0.37) |  | (0.19) | (0.24) |
| User-friendliness: high | 0.44* | -0.88** |  | 0.72*** | 0.05 |  | 0.49*** | 0.49* |
|  | (0.25) | (0.35) |  | (0.25) | (0.48) |  | (0.17) | (0.29) |
| Detailed output: moderate | 0.30 | 0.83** |  | 0.04 | 0.07 |  | 0.59*** | 0.04 |
|  | (0.22) | (0.34) |  | (0.23) | (0.60) |  | (0.17) | (0.41) |
| Detailed output: high | 0.27 | 0.46 |  | 0.37 | 0.42 |  | 0.31* | -0.11 |
|  | (0.22) | (0.45) |  | (0.24) | (0.46) |  | (0.17) | (0.39) |
| Predictive power | 0.01 | -0.00 |  | 0.01 | -0.00 |  | 0.01*** | 0.01*** |
|  | (0.00) | (0.01) |  | (0.00) | (0.01) |  | (0.00) | (0.00) |
| Platform: paper | -0.15 | 1.37*** |  | -0.22 | 0.16 |  | -0.29* | 0.65** |
|  | (0.23) | (0.33) |  | (0.22) | (0.92) |  | (0.16) | (0.28) |
| Platform: mobile | 0.63*** | 0.95*** |  | 0.40** | 0.47 |  | 0.42*** | -0.22 |
|  | (0.22) | (0.32) |  | (0.20) | (0.37) |  | (0.13) | (0.43) |
| Language: native | -0.15 | 0.94*** |  | 0.51** | -0.12 |  | 0.30* | 0.11 |
|  | (0.23) | (0.30) |  | (0.22) | (0.56) |  | (0.16) | (0.31) |
| Language: English+ native | 0.31 | 1.12*** |  | 0.59** | 0.61 |  | 0.39* | 0.73*** |
|  | (0.28) | (0.35) |  | (0.30) | (0.39) |  | (0.21) | (0.25) |
| N | 2196 |  |  | 1080 |  |  | 2484 |  |
| Log Likelihood | -511.85 |  |  | -245.15 |  |  | -564.492 |  |
| AIC | 1067.71 |  |  | 534.31 |  |  | 1172.99 |  |
| BIC | 1192.99 |  |  | 643.97 |  |  | 1300.97 |  |

***Notes***: Asterisks ***, **, and * denote any variable significant at 1%, 5%, and 10% levels respectively.

Standard errors reported between parentheses.

Table A4

Criteria for the selection of optimal number of preference classes (N=5760)

| Classes | Log-likelihood (LL) | # of parameters (k) | Akaike information criterion (AIC) | Consistent Akaike information criterion (CAIC) | Bayesian  information criterion (BIC) |
| --- | --- | --- | --- | --- | --- |
| 2 | -1344.04 | 23 | 2734.08 | 2910.23 | 2887.23 |
| 3 | -1321.49 | 35 | 2712.99 | 2981.03 | 2946.03 |
| 4 | -1308.49 | 47 | 2710.98 | 3070.94 | 3023.94 |
| 5 | -1293.10 | 59 | 2704.20 | 3156.06 | 3097.06 |

***Notes***: N is the number of observations on choice responses from 320 extension agents (3 alternatives × 6 choice sets × 320). The AIC is calculated as −2LL + 2k, the CAIC as −2LL + k ln(N+1), and the BIC as −2LL + k ln(N).

**Survey questionnaire**

# Taking Maize Agronomy to Scale in Africa (TAMASA)

**Extension Agents Survey 2016**

“We are part of a team at CIMMYT, IITA, KU Leuven Belgium, and Centre for Dryland Agriculture, Bayero University Kano, who are studying about agronomic advisory services for sustainable agricultural intensification in the country. Your participation in this survey is very much appreciated and your responses will be COMPLETELY CONFIDENTIAL. In addition, your responses will be added to those of ~300 other extension agents and analysed together. If you indicate your voluntary consent by participating in this interview, may we begin? If you have any questions or comments about this survey, you may contact Oyinbo Oyakhilomen, phone: +234-8032779442; ema[il: oyakhilomen.oyinbo@kuleuven.be.](mailto:oyakhilomen.oyinbo@kuleuven.be)”

#### SECTION 1: BASIC INFORMATION

| Extension agent name eaname | EA No. | eaid |
| --- | --- | --- |
| Mobile phone number phone | Date (dd/mm/yyyy) | surdate |

| **Identifying Variables:** | |  | | | | | | | | |
| --- | --- | --- | --- | --- | --- | --- | --- | --- | --- | --- |
| Supervisor: | | snum | | | | | | | | |
| Enumerator: | | enum | | | | | | | | |
| State: | | state | | | | | | | | |
| LGA under your coverage: | | lga | | | | | | | | |
| GPS coordinates: | Latitude (south) | ea_lat |  |  | **.** |  |  |  |  |  |
|  | Longitude (east) | ea_long |  |  | **.** |  |  |  |  |  |
| Altitude (m. a. s. l.) |  | ea_alt | | | | | | | | |

##### SECTION 2.1: CHOICE EXPERIMENT

Table 2.1: Responses to the choice experiment

| Choice task | Choice card identification number (1 to12) | Choice made (0 = Option C, 1 = Option A, 2 = Option B) |
| --- | --- | --- |
|  | **cn** | **cm** |
| 1 |  |  |
| 2 |  |  |
| 3 |  |  |
| 4 |  |  |
| 5 |  |  |
| 6 |  |  |

##### SECTION 2.2: Choice experiment accompanying questions

Table 2.2: Kindly indicate if there are one or more of the 6 attributes that you didn’t attend to or take into consideration in the choices you made

| S/No | Attributes | Attributes codes | Attribute non-attendance (1=yes, 2=no) |
| --- | --- | --- | --- |
|  |  | **att** | **ana** |
| 1 | Level of user-friendliness | a |  |
| 2 | Level of detailed output | b |  |
| 3 | Predictive power | c |  |
| 4 | Delivery platform | d |  |
| 5 | Delivery language | e |  |
| 6 | Time cost | f |  |

Table 2.3: Kindly indicate if there are one or more levels of the attributes that you didn’t attend to or take into consideration in the choices you made

| S/No | Attributes | Attributes codes | Attribute level non-attendance | | | | |  |
| --- | --- | --- | --- | --- | --- | --- | --- | --- |
|  |  | **att** | **alna1** | **alna2** | **alna3** | **alna4** | **alna5** | **alna6** |
| 1 | Level of user-friendliness | a |  |  |  |  |  |  |
| 2 | Level of detailed output | b |  |  |  |  |  |  |
| 3 | Predictive power | c |  |  |  |  |  |  |
| 4 | Delivery platform | d |  |  |  |  |  |  |
| 5 | Delivery language | e |  |  |  |  |  |  |
| 6 | Time cost | f |  |  |  |  |  |  |

| Attribute levels codes | | | | | |
| --- | --- | --- | --- | --- | --- |
| User-friendliness | Level of detailed output | Predictive power | Delivery platform | Delivery language | Time cost |
| 1- Low | 1- Low | 1- < 31% | 1. Non-mobile (desktops/laptops) 2. Quick guides (paper-based version) 3. Mobile (smartphones/tablets) | 1- English only | 1- 15 minutes |
| 2- Moderate | 2- Moderate | 2- 31 – 50% |  | 2- Native only | 2- 30 minutes |
| 3- High | 3- High | 3- 51 – 70% |  | 3- English + Native | 3- 45 minutes |
|  |  | 4- 71 – 90% |  |  | 4- 60 minutes |
|  |  | 5- > 90% |  |  |  |

Table 2.3: Kindly rank the attributes from the most important to the least important (Rank most important as 1 down to the least important as 6)

| S/No | Attributes | Attributes codes | Rank |
| --- | --- | --- | --- |
|  |  | **att** | **rk** |
| 1 | Level of user-friendliness | a |  |
| 2 | Level of detailed output | b |  |
| 3 | Predictive power | c |  |
| 4 | Delivery platform | d |  |
| 5 | Delivery language | e |  |
| 6 | Time cost | f |  |

Table 2.4: Kindly indicate your preference for some features to be included in a nutrient management decision support tool

| 1 | Most preferred delivery platform? 1= Quick guides (Paper version), 2= Non-mobile (desktops/laptops), 3= Mobile  (smartphones/tablets) | **pf1** |  |
| --- | --- | --- | --- |
| 2 | Most preferred delivery language? 1= English only, 2= Native only, 3= English + Native | **pf2** |  |
| 3 | Unit(s) of measurement you desire for maize output? 1= kg, 2= tonne, 3= 100 kg bag | **pf3** |  |
| 4 | Unit(s) of measurement you desire for inorganic fertilizer? 1= kg, 2= 50kg bag, 6=mudu, 7=tiya | **pf4** |  |
| 5 | Unit(s) of measurement you desire for organic fertilizer? 1= kg, 2= tonne, 3=cartload, 4=pickup, 5=mangala | **pf5** |  |

##### SECTION 3: DEMOGRAPHIC CHARACTERISTICS

Table 3: Demographic Characteristics

| 3.1 | What is your gender? *1=male, 2=female* | **gen** |  |
| --- | --- | --- | --- |
| 3.2 | What is your age? | **Ag** |  |
| 3.3 | What is your marital status? *1=single (never married) 2= monogamously married 3=polygamously married 4=divorced*  *5=widowed 6=separated* | **mstat** |  |
| 3.4 | What is your highest educational qualification? *1=pre-school, 2=Primary, 3=secondary, 4=OND, 5=HND, 6=NCE, 7=Degree,*  *8=Postgraduate* | **heq** |  |
| 3.5 | How many of your household members are adults (≥18 years to 65)? | **Adults** |  |
| 3.6 | How many of your household members are age 10-18? | **Child1015** |  |
| 3.7 | How many of your household members are below age 10? | **Childlt10** |  |
| 3.8 | How many of your household members are over age 65? | **Elders** |  |
| 3.9 | What organization are you affiliated to? *1=KTARDA, 2=KADP, 3=KNARDA, 4=Doreo partners, 5=Sasakawa, 6= KTARDA and*  *Sasakawa, 7= KADP and Sasakawa, 8= KNARDA and Sasakawa* | **extorg** |  |
| 3.10 | What is your employment status in your organization? *1=zonal extension officer/zonal director of extension, 2=area extension*  *officer/area project coordinator, 3=block extension officer, 4=village extension officer, 5= mik, 6=other, specify* | **empstat** |  |
| 3.11 | How long have you been working with an extension organization? | **Yrsofexp** |  |
| 3.12 | Do you engage in agricultural production? *1=yes, 2= no* | **agrprd** |  |
| 3.13 | If yes to Q3.12, Are you into maize production? *1=yes, 2= no* | **mprd** |  |
| 3.14 | How long have you been into maize production? | **Yrsofmprd** |  |

##### SECTION 4: INCOME SOURCES

Table 4: Estimated income from different sources

Indicate your estimated income from December 2015 and November 2016 (include in-kind receipts)

|  | Income source | Monthly income (*Naira*) | Number of months income was received |
| --- | --- | --- | --- |
|  | **source** | **othincome1** | **othincome2** |
| Salary | 1 |  |  |
| Income from crop sales | 2 |  |  |
| Income from livestock/livestock products sales | 3 |  |  |
| Off-farm income | 4 |  |  |
| Income from business | 5 |  |  |
| Rental income (income from renting out land) | 6 |  |  |
| Rental income (income from renting out buildings) | 7 |  |  |
| Remittances (*includes money sent home by people living outside the home*) | 8 |  |  |
| Donations/gifts | 9 |  |  |

##### SECTION 5: WORK ENVIRONMENT

Table 5: Work Environment

| 5.1 | How many villages are under your coverage? | **Vilcov** |  |
| --- | --- | --- | --- |
| 5.2 | How many farmer groups are under your coverage? | **Fgrpcov** |  |
| 5.3 | How many farmers are under your coverage? | **Farcov** |  |
| 5.4 | Do you own an android phone? *1=yes, 2= no* | **oad** |  |
| 5.5 | Do you own a tablet? *1=yes, 2= no* | **otab** |  |
| 5.6 | Are you proficient in the use of android devices such as tablets, smart phones? *1= proficient, 2= not proficient* | **pad** |  |
| 5.7 | Are you currently using android devices such as tablets, smart phones in extension service delivery? *1=yes, 2= no* | **cuad** |  |
| 5.8 | Did you undergo any in-service training in the last one year? *1=yes, 2= no* | **ist** |  |
| 5.9 | If yes to Q5.8, how many in-service trainings have you undergone in the last one year? | **Noist** |  |
| 5.10 | If yes to Q5.8, how many of the training(s) had topic on fertilizer recommendation and/or soil fertility related issues? | **Frist** |  |
| 5.11 | If yes to Q5.7, how many of the training(s) were on the use of computer devices such as tablets, smart phones to disseminate  extension messages to farmers? | **Cdist** |  |
| 5.12 | How many hours do you work in a day? | **Hrsdy** |  |
| 5.13 | What percentage of your total working time do you spend giving farmers advice on soil fertility management?  *1=10%, 2=20%, 3=30%, 4=40%, 5-50%, 6=60%, 7=70%, 8=80%, 9=other, specify* | **pttsfm** |  |
| 5.14 | Do you have access to bicycle, motorcycle or vehicle from your organization to facilitate your movements in reaching out to farmers? | **Bmv** |  |
| 5.15 | Do you get your promotions as at when due/regular promotion? *1=yes, 2= no* | **prom** |  |
| 5.16 | Do you get timely payment of your salary/allowances? *1=yes, 2= no* | **sal** |  |
| 5.17 | Do you get adequate supervision from your superiors? *1=yes, 2= no* | **super** |  |
| 5.18 | Do you get adequate training opportunities from your organization? *1=yes, 2= no* | **tropp** |  |
| 5.19 | What is your perception of your job security? *1= secured, 2=not secured* | **jbsec** |  |
| 5.20 | What is your perception of your job satisfaction as an extension officer? *1= very high satisfaction, 2=high satisfaction, 3=moderate*  *satisfaction, 4= low satisfaction, 5= very low satisfaction* | **jbsat** |  |

##### SECTION 6: FERTILIZER RECOMMENDATIONS

Table 6: Fertilizer Recommendations

| 6.1 | Are you aware of the concept of field/site-specific fertilizer recommendations? *1=yes, 2= no* | **fr1** |  |
| --- | --- | --- | --- |
| 6.2 | If yes to Q6.1, what is your main source of awareness of field/site-specific fertilizer recommendations? *1= educational background 2=training*  *3=fellow extension agents 4=agrodealers 5= radio 6=television 7 = print media (pamphlets, bulletins, posters) 8= others specify* | **fr2** |  |
| 6.3 | How often do farmers seek advice on fertilizer recommendation from you? *1= very often 2=often 3=not often* | **fr3** |  |
| 6.4 | Have you been involved in disseminating information on fertilizer recommendation to maize-based farmers? *1=yes, 2= no* | **fr4** |  |
| 6.5 | If yes to Q7.1, for how long have you been disseminating information on fertilizer recommendation to maize-based farmers? | **Fr5** |  |
| 6.6 | What fertilizer recommendation do you prefer to give farmers? *1=recommendations with point estimate of attainable yield (e.g apply a specific*  *NPK rate and get 40 bags of maize per hectare) 2=recommendations with interval estimate of attainable yield (e.g apply a specific NPK rate and get 37 to 42 bags of maize per hectare)* | **fr6** |  |

##### SECTION 7: TIME AND RISK PREFERENCES

Table 7: Time and risk preference

Kindly respond to the following hypothetical choice decisions. There is no right or wrong decision.

| 7.1 | If you were to be offered allowance for outstanding extension service, would you prefer to receive 40,000 Naira now or 50,000 Naira one  month from now? *1=40,000 Naira now, 2=50,000 Naira in 1 month* | **t1** |  |
| --- | --- | --- | --- |
| 7.2 | If you were to be offered allowance for outstanding extension service, would you prefer to receive 40,000 Naira now or 55,000 Naira two  months from now? *1=40,000 Naira now, 2=55,000 Naira in 2 months* | **t2** |  |
| 7.3 | If you were to be offered allowance for outstanding extension service, would you prefer to receive 40,000 Naira now or 65,000 Naira three  months from now? *1=40,000 Naira now, 2=65,000 Naira in 3 months* | **t3** |  |
| 7.4 | If you were to be offered allowance for outstanding extension service, would you prefer to receive 40,000 Naira now or 75,000 Naira four  months from now? *1=40,000 Naira now, 2=75,000 Naira in 4 months* | **t4** |  |
| 7.5 | If you were to be offered allowance for outstanding extension service, would you prefer to receive 40,000 Naira now or 80,000 Naira five  months from now? *1=40,000 Naira now, 2=80,000 Naira in 5 months* | **t5** |  |
| 7.6 | Consider four possible outcomes of a hypothetical investment of 120,000 Naira: 1=50% chance of gaining 200,000 Naira and a 50% chance of losing 100,000 Naira. 2=50% chance of gaining 190,000 Naira and a 50% chance of losing 70,000 Naira. 3=50% chance of gaining 180,000 Naira and a 50% chance of losing 50,000 Naira. 4=50% chance of gaining 160,000 Naira and a 50% chance of losing 40,000 Naira. 5=50% chance of gaining 150,000 Naira and a 50% chance of losing 30,000 Naira.  *Which of these 5 options would you choose?* | **T6** |  |

##### SECTION 8: MULTIDIMENTIONAL POVERTY INDICATORS

Table 8: Multidimensional Poverty Indicators

| Nutrition | have at least 1 household member who is malnourished | *1=yes, 2=no* | **mpi1** |  |
| --- | --- | --- | --- | --- |
| Child mortality | have experienced child mortality in the past 5 years | *1=yes, 2=no* | **mpi2** |  |
| Years of schooling | have no household member who completed at least 5 years of schooling | *1=yes, 2=no* | **mpi3** |  |
| Child school attendance | have at least 1 school-aged child not attending school | *1=yes, 2=no* | **mpi4** |  |
| Electricity | have access to electricity | *1=yes, 2=no* | **mpi5** |  |
| Cooking fuel | cook with wood or charcoal | *1=yes, 2=no* | **mpi6** |  |
| Toilet | have adequate sanitation | *1=yes, 2=no* | **mpi7** |  |
| Drinking water | have access to clean drinking water | *1=yes, 2=no* | **mpi8** |  |
| Floor | have a sand floor in the house | *1=yes, 2=no* | **mpi9** |  |
| Assets | own more than one of the following assets; radio, TV, telephone, bike, motorbike or refrigerator  and own a car or truck | *1=yes, 2=no* | **mpi10** |  |

##### SECTION 9: FOOD SECURITY

Table 9: Household dietary diversity

Kindly Indicate the foods that you or any member of your household ate or drank in the last 24 hours (24 hours recall period), whether at home or outside the home

| Cereal: maize, rice, wheat, sorghum, millet or any other grains or foods made from these (e.g. bread, noodles, porridge etc.) | *1=yes, 2=no* | **Fs1** |  |
| --- | --- | --- | --- |
| White root and tubers: white potatoes, white yam, white cassava, cocoyam or other foods made from roots | *1=yes, 2=no* | **fs2** |  |
| Vitamin A rich vegetables and tubers: pumpkin, carrot, squash, or sweet potato that are orange inside + other locally available  vitamin A rich vegetables (e.g. red sweet pepper) | *1=yes, 2=no* | **fs3** |  |
| Dark green leafy vegetables: dark green leafy vegetables, including wild forms + locally available vitamin A rich leaves such as  amaranth, cassava leaves, spinach, etc. | *1=yes, 2=no* | **fs4** |  |
| Other vegetables: other vegetables (e.g. tomato, onion, eggplant) + other locally available vegetables | *1=yes, 2=no* | **fs5** |  |
| Vitamin A rich fruits: orange, mango, pawpaw, guava, pine apple, water melon, apricot (fresh or dried) and 100% fruit juice made  from these + other locally available vitamin A rich fruits, etc. | *1=yes, 2=no* | **fs6** |  |
| Other fruits: other fruits, including wild fruits and 100% fruit juice made from these | *1=yes, 2=no* | **fs7** |  |
| Organ meat: liver, kidney, heart or other organ meat or blood-based foods | *1=yes, 2=no* | **fs8** |  |
| Flesh meat: beef, chicken, pork, lamb, goat, rabbit, duck, other birds, etc. | *1=yes, 2=no* | **fs9** |  |
| Egg: eggs from chicken, duck, guinea fowl or any other egg | *1=yes, 2=no* | **fs10** |  |
| Fish and sea food: fresh or dried fish or shellfish | *1=yes, 2=no* | **fs11** |  |
| Legumes and nuts: cowpea, soybean, bambara nut, dried beans, dried peas, lentils, nuts, seeds or foods made from these (e.g.  Groundnut cake, peanut butter) | *1=yes, 2=no* | **fs12** |  |
| Oil and fats: oil, fats or butter added to food or used for cooking | *1=yes, 2=no* | **fs13** |  |
| Sweets: sugar, honey, sweetened soda or sweetened juice drinks, sugary foods | *1=yes, 2=no* | **fs14** |  |
| Spices, condiments and beverages: spices (black pepper, salt), condiments (soy sauce, hot sauce), coffee, tea | *1=yes, 2=no* | **fs15** |  |

**Thank you very much for participating in this survey and for your time!**
